# Psychiatric Comorbidity in a Neuropsychiatric Patient Cohort

**DOI:** 10.1192/bjo.2026.11108

**Published:** 2026-06-30

**Authors:** Leonidas Nihoyannopoulos, Bruce Tamilson

**Affiliations:** South West London and St. George’s NHS Mental Health Trust, London, United Kingdom

## Abstract

**Aims:**

Patients with neurological disorders frequently present with co-occurring psychiatric symptoms, often requiring assessment within specialist neuropsychiatric services. This study aimed to describe the spectrum of psychiatric presentations seen in a specialist neuropsychiatry outpatient population and evaluate the prevalence and severity of anxiety and depressive symptoms among patients attending a tertiary neuropsychiatry clinic.

**Methods:**

A descriptive cross-sectional study was conducted in a tertiary regional neuropsychiatry outpatient clinic in London. Consecutive patients attending the service were invited to participate. Participants completed a standardised questionnaire collecting demographic data and standardised measures of mood and anxiety were administered using the Patient Health Questionnaire-9 (PHQ-9) and the Generalised Anxiety Disorder-7 (GAD-7). Of those invited, 79 participants were included in the final analysis.

**Results:**

High levels of psychiatric comorbidity were observed across the neuropsychiatric cohort, with substantial variability between diagnostic groups. Patients diagnosed with functional neurological disorder (FND) demonstrated the greatest affective symptom burden: 92% reported at least mild depressive symptoms and 83% met criteria for clinically significant anxiety. Other neuropsychiatric subgroups similarly exhibited notable levels of anxiety and depression although to a lesser extent than the FND group.

**Conclusion:**

This study demonstrates a substantial and heterogeneous psychiatric symptom burden among patients attending a specialist neuropsychiatry outpatient service. The particularly high prevalence of affective symptoms in individuals with FND highlights the need for comprehensive, integrated, and multidisciplinary approaches to assessment and treatment. Improved recognition and management of psychiatric comorbidities may have important implications for clinical outcomes, patient experience, and long-term prognosis within neuropsychiatric populations.

